# Cluster of Differentiation 36-Mediated Inflammation and Lipid Metabolism in Cardiovascular Diseases: From Mechanisms to Novel Therapies

**DOI:** 10.3390/antiox15060694

**Published:** 2026-05-30

**Authors:** Jiayin Song, Xiangnuo Han, Yu Zhang, Meixiu Jiang

**Affiliations:** 1 The Queen Mary School, Jiangxi Medical College, Nanchang University, 999 Xuefu Road, Nanchang 330031, China; 2 Jiangxi Province Key Laboratory of Bioengineering Drugs, The National Engineering Research Center for Bioengineering Drugs and the Technologies, Institute of Translational Medicine, Jiangxi Medical College, Nanchang University, 999 Xuefu Road, Nanchang 330031, China; 3 Institute of Biomedical Innovation, Jiangxi Medical College, Nanchang University, 999 Xuefu Road, Nanchang 330031, China

**Keywords:** CD36, inflammation, lipid metabolism, cardiovascular disease, therapy

## Abstract

Cardiovascular diseases (CVDs) continue to pose a key challenge to public health because of their high prevalence, mortality, and disability rates, together with a trend toward younger age at onset. Chronic inflammation and disordered lipid metabolism are key pathological mechanisms underlying the development and progression of CVDs. Although numerous pharmacological agents have been developed to intervene in CVDs, current therapies are still limited by adverse effects, suboptimal efficacy, and insufficient anti-inflammatory properties. Consequently, effective drug-based strategies for the prevention and treatment of certain CVDs are still insufficient. Cluster of differentiation 36 (CD36), a class B scavenger receptor, mediates the recognition and uptake of long-chain fatty acids, oxidized low-density lipoprotein, and other ligands, and is present in diverse tissues and cell types. Accumulating evidence indicates that CD36 plays a critical role in lipid metabolism and inflammatory signalling pathways implicated in CVDs, suggesting that it represents a promising focus for treatment-oriented interventions. This review synthesizes current evidence on the multifaceted roles of CD36 in lipid metabolism dysregulation and inflammation-associated pathways in CVDs and evaluates its potential as a tractable target for disease prevention and management.

## 1. Introduction

Cardiovascular diseases (CVDs) comprise a heterogeneous group of diseases affecting the cardiovascular system, including cardiomyopathy, valvular heart disease, coronary artery disease (CAD), stroke, peripheral artery disease, heart failure (HF), and arrhythmias. Data from the World Health Organization (WHO) indicates that CVDs constitute the foremost cause of global mortality, claiming nearly 18 million deaths annually and more than 31% of all global deaths [1]. Atherosclerosis represents the most common pathological basis of most CVDs [2], and atherosclerotic cardiovascular diseases remain the leading cause of mortality among all CVD subtypes [3]. The worldwide impact of CVDs continues to increase, accompanied by an alarming trend toward younger age at onset [4,5]. In addition, major cardiovascular events such as myocardial infarction and stroke often result in severe long-term sequelae, including hemiplegia, aphasia, cognitive impairment, and loss of working capacity [6,7]. The high costs of medication, surgery, rehabilitation, and prolonged care, as well as productivity loss secondary to disability, impose a considerable financial burden on households and healthcare systems worldwide.

The etiopathology of CVDs is mainly associated with metabolic disorders, especially lipid deposition during atherosclerotic plaque formation [8]. Nevertheless, evidence indicating that lipid deposition and metabolic dysregulation cause active inflammatory reactions in the vascular wall has steadily mounted over the last 20 years in basic and clinical investigations [9]. Therefore, atherosclerosis is no longer regarded as a simple process of passive lipid accumulation. It is now recognized as a chronic and progressive inflammatory disease initiated by endothelial damage and powered by multiple cardiovascular risk factors. In this context, recruitment and activation of immune cells—together with the secretion of pro-inflammatory mediators such as tumour necrosis factor-α (TNF-α), interleukin (IL)-1β, and IL-6—collectively promote plaque initiation, progression, destabilization, and rupture, precipitating life-threatening events in the form of stroke or CAD [10]. Beyond atherosclerosis, chronic inflammation also significantly contributes to the pathogenesis of hypertension, HF, atrial fibrillation, aortic aneurysm, and aortic dissection [11,12,13].

Current pharmacological strategies for CVDs mainly focus on reducing blood pressure, lipid profile improvement, antithrombotic treatment, relief of angina, and control of HF or arrhythmias. Representative drug classes include angiotensin-converting enzyme inhibitors (ACEIs), angiotensin II receptor blockers (ARBs), calcium channel blockers (CCBs), β-blockers, mineralocorticoid receptor antagonists (MRAs), aspirin, statins, and warfarin. These therapies are indispensable, but they do not eliminate cardiovascular risk. Even among patients receiving optimal guideline-directed therapy and achieving recommended low-density lipoprotein cholesterol (LDL-C), blood pressure, and antithrombotic targets, recurrent cardiovascular events remain common [14]. This unmet need is now conceptualized as residual cardiovascular risk, which may arise from residual cholesterol risk, triglyceride, lipoproteins, diabetes-related metabolic injury, thrombotic tendency, and persistent vascular inflammation. Among these components, residual inflammatory risk has received increasing attention because it may exacerbate plaque vulnerability, which increases the probability of plaque rupture and subsequent thrombogenesis [15]. Therefore, a deeper understanding of inflammatory mechanisms in CVDs and the discovery of new therapeutic targets are still key issues in cardiovascular studies.

Cluster of differentiation 36 (CD36), also known as scavenger receptor class B type 2, is a multifunctional receptor functioning as a pattern-recognition receptor for damage-associated molecular patterns (DAMPs), pathogen-associated molecular patterns (PAMPs), and a transporter for a variety of lipids and proteins. CD36 is a commonly expressed glycosylated membrane protein of approximately 80 kDa present in numerous cell types, including platelets, macrophages, monocytes, adipocytes, myocytes, enterocytes, enteroendocrine cells, epithelial cells, and endothelial cells [16,17]. Structurally, CD36 contains a large glycosylated extracellular domain with two transmembrane domains and short cytoplasmic tails at both termini, forming a characteristic hairpin-like topology [16]. CD36 is increasingly recognized as a critical mediator linking lipid metabolic dysregulation to chronic inflammation. By facilitating the utilization of oxidized low-density lipoprotein (oxLDL) and sensing abnormal metabolic signals, CD36 promotes foam cell formation [18] and activates other downstream inflammatory pathways, such as the NOD-like receptor pyrin domain-containing 3 (NLRP3) inflammasome, thus contributing to persistent inflammatory responses and disease progression [19]. In sum, this makes CD36 a central connection underlying the interplay between disordered lipid metabolism and chronic inflammation, highlighting its promise as an intervention target for chronic inflammatory and lipid metabolism-related diseases.

**Figure 1:**
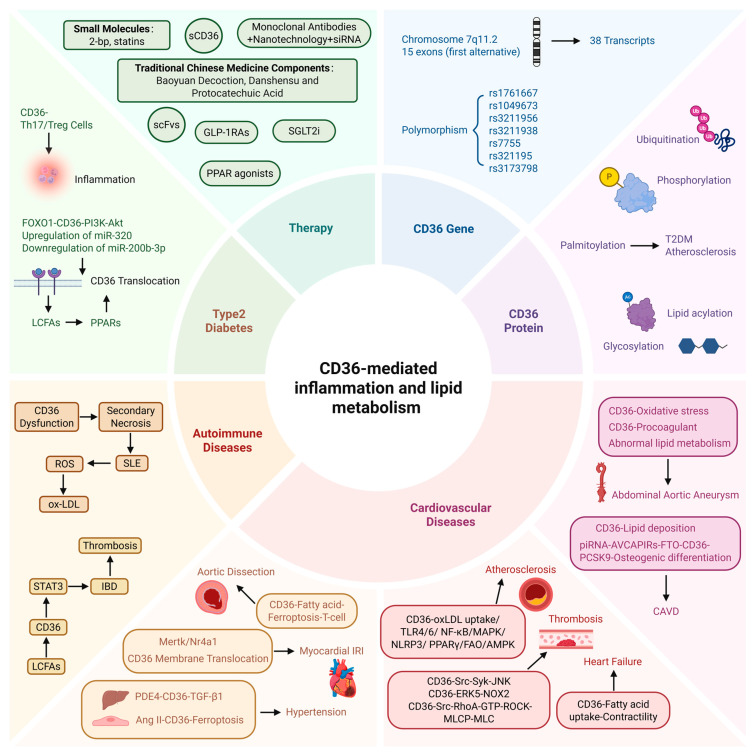
CD36-mediated inflammation and lipid metabolism. Polymorphisms of the CD36 gene and post-translational modifications of its protein are associated with susceptibility to cardiovascular diseases (CVDs). CD36 participates in lipid metabolism and inflammatory responses in CVDs, as well as in cardiovascular complications of autoimmune diseases and diabetes. CD36—cluster of differentiation 36; CAVD—calcific aortic valve disease; piRNA-AVCAPIRs—aortic valve calcification-associated PIWI-interacting RNA; FTO—fat mass and obesity-associated protein; PCSK9—proprotein convertase subtilisin/kexin type 9; oxLDL—oxidized low-density lipoprotein; TLR—Toll-like receptor; NF-κB—nuclear factor κB; MAPK—mitogen-activated protein kinase; NLRP3—NOD-like receptor pyrin domain-containing 3; PPARγ—peroxisome proliferator-activated receptor γ; FAO—fatty acid oxidation; AMPK—AMP-activated protein kinase; JNK-c—Jun N-terminal kinase; ERK5—extracellular signal-regulated kinase 5; NOX2—NADPH oxidase-2; ROCK—RhoA kinase; MLCP—myosin light chain phosphatase; Mertk—myeloid epithelial reproductive receptor tyrosine kinase; Nr4a1—nuclear receptor subfamily 4, group A, member 1; PDE4—phosphodiesterase 4; TGF-β—transforming growth factor-β1; Ang II—angiotensin II; IRI—ischaemia–reperfusion injury; IBD—inflammatory bowel disease; LCFAs—long-chain fatty acids; STAT3—signal transducer and activator of transcription 3; ROS—reactive oxygen species; SLE—systemic lupus erythematosus; FOXO1—forkhead box protein O1; PI3K-AKT—phosphatidylinositol 3-kinase-Akt; CVDs—cardiovascular diseases. Created in BioRender. Song, J. (2026), https://BioRender.com/g8l3065, accessed on 14 May 2026.

The present review summarizes the role of CD36 in lipid metabolism and inflammatory signalling and its role in CVDs, as well as cardiovascular complications secondary to inflammatory diseases. We further highlight advances in CD36-targeted therapeutic strategies for CVDs, emphasizing their translational potential and challenges. By integrating and analysing the available evidence, this review aims to identify existing knowledge gaps and provide insights to guide future mechanistic and translational research (Figure 1).

## 2. CD36 Gene and Post-Translational Modifications

### 2.1. CD36 Gene and Gene Polymorphism

**Figure 2:**
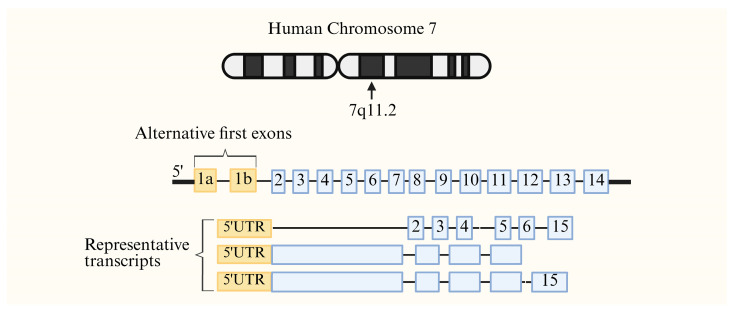
The chromosomal location and specific expression pattern of the CD36 gene. The CD36 gene is located in the 7q11.2 region of chromosome 7 and comprises 15 exons; the first exon is associated with a tissue-specific promoter. Created in BioRender. Song, J. (2026), https://BioRender.com/spim0dk, accessed on 15 May 2026.

The CD36 gene is situated on chromosome 7q11.2 and contains 15 exons [20]. The CD36 gene is characterized by alternative first exons, each of which is regulated by its own independent promoter. The tissue-specific alternative splicing of the first exon in platelets, monocytes, macrophages, endothelial cells, adipocytes, dendritic cells, muscle cells, liver, and haematopoietic cells reflects pronounced promoter specificity and allows the CD36 gene to generate up to 38 distinct transcripts (Figure 2).

The CD36 gene polymorphism has always been of great concern. Accumulating evidence indicates that CD36 genetic variants correlate with CVDs and that the distribution and effects of these variants may differ across ethnic populations. For example, *rs1761667* has been identified as a susceptibility locus for hypertension in an Iranian population [21]. In addition, carriers of the A allele at the *rs1761667* locus are at increased risk of early-onset coronary artery disease (EOCAD), with this association being more pronounced in Caucasian and Chinese populations [22]. Notably, patients carrying the *AG* genotype exhibited significantly elevated plasma oxLDL levels compared with individuals harbouring either the *GG* or *AA* genotype (*p* = 0.010) [23]. By contrast, carriers of the *G* allele at the *rs1049673* and *rs3211956* loci appear to have a lower risk of EOCAD, and this protective, anti-atherosclerotic effect is more evident in the Chinese population [22]. Importantly, the influence of CD36 variants on EOCAD risk appears to be mediated primarily through dyslipidaemia. In addition, among African American individuals, the *G* allele of *rs3211938* has been reported to be more susceptible to dilated cardiomyopathy [24]. Sex-specific effects of CD36 polymorphisms have also been reported. In northern Chinese Han populations, *rs1049673, rs7755, rs321195,* and *rs3173798* were significantly linked to atherosclerotic risk in men, whereas no significant association was observed in women [25].

Collectively, these findings suggest that CD36 polymorphisms may influence cardiovascular susceptibility through complex interactions involving lipid metabolism, ethnicity, and sex.

### 2.2. Distribution of CD36 Protein

CD36 is distributed among various cell types and performs distinct biological functions depending on the cellular context. In macrophages, it is abundantly expressed and plays a key role in recognizing and internalizing apoptotic cells and oxidized lipoproteins, thereby promoting foam cell formation and atherosclerosis progression [26]. CD36 is also present in monocytes and dendritic cells, where it participates in antigen recognition and inflammatory signalling [27]. In vascular endothelial cells, CD36 is abundantly expressed and primarily facilitates the uptake of long-chain fatty acids (LCFAs), thus providing energy substrates for highly oxidative tissues such as the heart and skeletal muscle [28]. In adipocytes, CD36 facilitates fatty acid uptake and triglyceride synthesis for lipid storage [29]. In addition, CD36 is present on the surface of platelets, where, as a receptor for thrombosis-associated ligands, it contributes to platelet adhesion and recruitment and subsequent thrombus development following atherosclerotic plaque rupture [30].

### 2.3. Post-Translational Modification of CD36

Post-translational regulation of CD36 includes palmitoylation, ubiquitination, phosphorylation, glycosylation, and lipid acylation [29,31,32]. Increasing evidence suggests that these modifications are closely associated with cellular metabolic status and inflammatory responses, with palmitoylation attracting particular attention. Dynamic palmitoylation precisely regulates the membrane localization, lipid raft stability, and endocytic trafficking of CD36 through an enzymatic network involving DHHC family acyltransferases, such as DHHC4 and DHHC5, as well as the depalmitoylase APT1 [33,34]. Palmitoylation increases CD36 in lipid raft microdomains, enhances its functional cooperation with receptors such as Toll-like receptor (TLR) 4, and thereby augments fatty acid uptake and inflammatory signalling, including NLRP3 inflammasome assembly and the release of pro-inflammatory mediators such as IL-1β [35]. This mechanism plays a central role in the pathogenesis of metabolic diseases such as type 2 diabetes (T2DM) and atherosclerosis, in part by reinforcing a vicious cycle between lipid overload and chronic inflammation. Accordingly, targeting CD36 palmitoylation represents a promising therapeutic approach for metabolic disorders, with considerable translational potential.

## 3. CD36 and Cardiovascular Diseases

Evidence indicates that CD36 is implicated in the onset and progression of different CVDs, with its role in atherosclerosis being the most commonly researched. The mechanisms by which CD36 regulates inflammation and lipid metabolic dysregulation in atherosclerosis are fairly well understood. CD36 has been reported to be involved in other CVDs, such as hypertension, abdominal aortic aneurysm, aortic dissection, calcific aortic valve disease, and ischaemia–reperfusion injury. Nevertheless, because of the functional complexity of CD36 and its cell-dependent effects, the underlying mechanistic network remains incompletely understood. This review incorporates the existing evidence by summarizing the role of CD36 in CVDs, highlighting key directions for future mechanistic studies, and evaluating the potential of precise CD36-targeted strategies for disease prevention and treatment. Notably, several autoimmune diseases have also been shown to increase CVD risk, and CD36 may represent an important mechanistic link between these conditions.

### 3.1. CD36 and Hypertension

Hypertension ranks among the most prevalent chronic health conditions, and its worldwide prevalence is rising. This disease is typically asymptomatic early on, but it continually inflicts damage to vital organs, leading to atherosclerosis, CAD, HF, stroke, and renal failure, among other serious cardiovascular, cerebrovascular, and renal complications [36]. Current therapeutic strategies for hypertension primarily rely on diuretics, ACEI, ARB, CCB, and β-blockers. Even though these agents are generally effective, managing hypertension remains challenging. Long-term daily medication is required, which might decrease patient adherence, while drug-related adverse effects can impair quality of life. Furthermore, the high degree of intersubjectivity in the response to drugs and suboptimal outcomes in refractory hypertension further limit therapeutic efficacy. Recent studies have highlighted a link between CD36 and hypertension involving multiple mechanisms, including lipid metabolism disorders, vascular endothelial dysfunction, and disruption of blood pressure circadian rhythm.

Non-alcoholic fatty liver disease (NAFLD) shows a pronounced relationship with hypertension. Under a high-fat diet or certain pathological conditions, hepatic CD36 can be excessively activated by phosphodiesterase 4 (PDE4), which not only aggravates lipid accumulation in the liver but also boosts the synthesis of transforming growth factor-β1 (TGF-β1). Elevated circulating TGF-β1 subsequently acts on the vasculature, inducing vascular inflammation and fibrosis, which promotes vascular stiffening and increased peripheral resistance, ultimately contributing to the development of hypertension [37]. CD36 also mediates hypertension associated with vascular endothelial injury. The latest research has demonstrated that angiotensin II (Ang II), a key pathogenic factor in hypertension, can stimulate CD36, initiate lipid peroxidation in vascular endothelial cells, and induce ferroptosis. This process compromises endothelial integrity, reduces the generation of vasodilatory factors such as nitric oxide (NO), and directly contributes to vascular dysfunction and elevated blood pressure [38].

Furthermore, CD36 gene polymorphisms may influence the occurrence and evolution of hypertension. Clinical studies confirm that CD36 mutations might interfere with the blood pressure circadian rhythm. High–normal blood pressure carriers with CD36 gene defects have significantly elevated nighttime systolic blood pressure and 24 h mean arterial pressure and have a higher incidence of the so-called non-dipper blood pressure pattern [39]. Under physiological conditions, blood pressure typically declines at night (dipper pattern); however, an attenuated nocturnal decline (non-dipper pattern) is associated with sustained hemodynamic stress on the cardiovascular system and accelerated target organ damage [40]. Nevertheless, the exact pathways through which CD36 genetic alterations contribute to hypertension have yet to be fully understood and warrant further investigation.

To sum up, CD36 gene polymorphisms affect blood pressure rhythmicity, while CD36 activation caused by factors such as PDE4 and Ang II promotes inflammatory responses and lipid metabolism disorders that contribute to the onset of hypertension. Collected data points to CD36 as a candidate therapeutic target for hypertension.

### 3.2. CD36 and Atherosclerosis/Thrombosis

Atherosclerosis is a chronic systemic vascular disorder characterized by persistent lipid deposition, foam cell accumulation, arterial wall inflammation, and thrombotic complications. Atherosclerotic cardiovascular diseases affect numerous individuals worldwide, with a significant number of new occurrences reported every year [41,42], representing the common pathological basis for ischemic stroke, coronary heart disease (CHD), and peripheral artery disease. Among these, CHD represents the foremost cause of death globally, followed by stroke in second place [43]. The complications of CHD, such as angina pectoris, myocardial infarction, HF, arrhythmias, and sudden death, have increased the complexity of this disease [44]. Moreover, a high rate of recurrences and disability linked with CHD and stroke greatly degrade the quality of life of patients, and treatment and rehabilitation expenses constitute a considerable economic burden [6]. However, even though standardization of this treatment course with lipid-lowering medications and antiplatelets has proven to be effective, the problem of drug side effects and compliance with therapy remains a serious concern. In particular, residual inflammatory risk has emerged as a critical limitation of current therapeutic strategies. Atherosclerosis is fundamentally a chronic inflammatory disease, and inflammation is a key driver in the formation, progression, and rupture of plaques. Notably, recent anti-inflammatory strategies have provided clinical evidence supporting inflammation as a therapeutic target in atherosclerotic cardiovascular disease. Low-dose colchicine can reduce cardiovascular events in patients with recent myocardial infarction and chronic coronary disease [45,46]. Furthermore, canakinumab, a monoclonal antibody targeting interleukin-1β, provides proof that selectively inhibiting inflammatory pathways can reduce recurrent atherothrombotic events in patients with previous myocardial infarction and elevated inflammatory markers [47]. Nevertheless, the clinical use of inflammation-targeted therapy is still limited by issues such as patient selection, safety, cost, and long-term benefit–risk balance. Therefore, further exploration of precise, safe, and effective anti-inflammatory strategies remains essential for improving the prevention and treatment of atherosclerosis.

**Figure 3:**
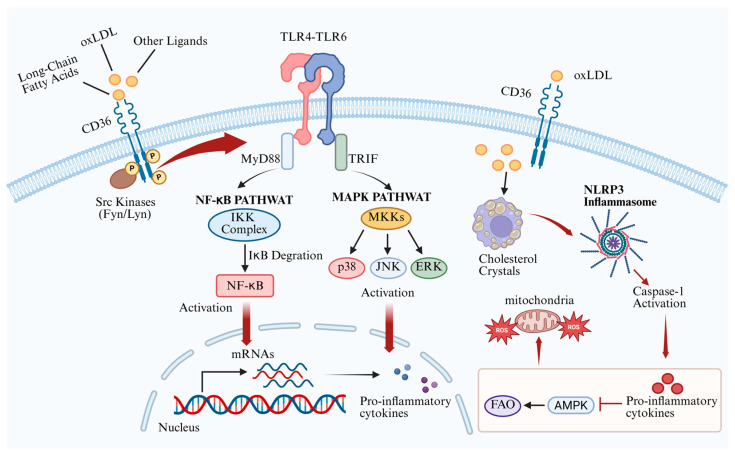
Regulatory mechanisms of macrophage CD36 in atherosclerosis. CD36 mediates the uptake of oxLDL and simultaneously activates the MAPK and NF-κB pathways, forming a pro-inflammatory loop. In addition, CD36 promotes foam cell formation, which activates the NLRP3 inflammasome, ultimately increasing ROS production. CD36—cluster of differentiation 36; oxLDL—oxidized low-density lipoprotein; TLR—Toll-like receptor; MyD88—myeloid differentiation primary response protein 88; TRIF—TIR domain-containing adaptor inducing interferon-β; NF-κB—nuclear factor κB; MAPK—mitogen-activated protein kinase; ERK—extracellular signal-regulated kinase; NLRP3—NOD-like receptor pyrin domain-containing 3; IL-1β—interleukin-1β; AMPK—AMP-activated protein kinase; FAO—fatty acid oxidation; ROS—reactive oxygen species. Cross symbols denote inhibition. Created in BioRender. Song, J. (2026) https://BioRender.com/mlx3314, accessed on 16 May 2026.

In 2000, Febbraio et al. found that knockout of CD36 in the apolipoprotein E gene-deficient (ApoE^−/−^) background contributed to a 70% reduction in plaque area, which was the first time CD36 was confirmed as a key gene promoting plaque formation [48]. Previous studies have demonstrated that CD36 is highly expressed in human atherosclerotic lesions, especially in the advanced stages of atherosclerosis [49]. The subendothelial retention and oxidation of modified LDL within the arterial intima are pivotal processes in the development and destabilization of atherosclerotic plaques [42]. Expressed on macrophages, CD36 accelerates atherosclerotic progression by recognizing and internalizing oxLDL, activating inflammatory pathways, and facilitating foam cell formation (Figure 3).

In macrophages, following the uptake of oxLDL, CD36 interacts with Src kinase and mediates the establishment of the TLR4–TLR6 complex through its intracellular C-terminal domain. Upon complex formation, TLRs engage with a series of adaptor proteins, such as myeloid differentiation primary response protein 88 (MyD88) and TIR domain-containing adaptor inducing interferon-β (TRIF), to trigger a series of reactions, thereby activating the mitogen-activated protein kinase (MAPK) and nuclear factor κB (NF-κB) pathways, which enter the nucleus to regulate factors such as activator protein 1 (AP-1), interferon regulatory factors (IRFs), and other pro-inflammatory cytokines [50]. Furthermore, continuous internalization of oxLDL through CD36 promotes the formation and accumulation of intracellular cholesterol crystals, driving foam cell formation. Nagy et al. provided evidence that oxLDL activates peroxisome proliferator-activated receptor (PPAR) γ through its oxidized lipid components, upregulating CD36 expression in macrophages and establishing a self-reinforcing loop that further promotes foam cell formation [51]. The imbalance between free cholesterol and cholesteryl esters within foam cells can activate the NLRP3 inflammasome, thereby promoting the maturation and release of IL-1β [52]. CD36 not only internalizes lipids but also impedes the reverse migration of cells, thereby confining the pro-inflammatory macrophage phenotype within the plaque and perpetuating chronic inflammation [53]. The inflammatory environment within foam cells suppresses AMP-activated protein kinase (AMPK) activity, impairing fatty acid oxidation (FAO) and promoting a shift to glycolytic metabolism. This metabolic alteration results in excessive production of reactive oxygen species (ROS), further sustaining NF-κB activation [52,54,55,56].

**Figure 4:**
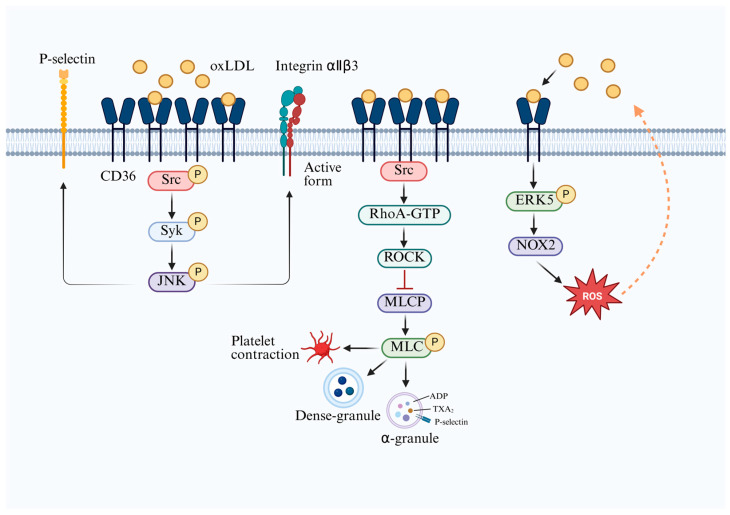
Regulatory mechanisms of platelet CD36 in thrombosis. The oxLDL–CD36 complex induces sequential phosphorylation of Src, Syk, and JNK, leading to activation and surface expression of P-selectin, thereby promoting thrombosis. CD36 also increases NOX2 activity via ERK5, generating ROS and producing more oxLDL, establishing a positive feedback loop that promotes atherosclerosis. CD36–Src complex facilitates RhoA-GTP formation, activates ROCK, inhibits MLCP, and ultimately triggers platelet contraction and filopodia extension, resulting in the release of ADP, TXA_2_, and P-selectin, thereby enhancing platelet aggregation and thrombus formation. CD36—cluster of differentiation 36; oxLDL—oxidized low-density lipoprotein; ROCK—RhoA kinase; MLCP—myosin light chain phosphatase; MLC—myosin light chains; ERK5—extracellular signal-regulated kinase 5; NOX-2—NADPH oxidase-2; ROS—reactive oxygen species; TXA2—thromboxane A_2_. Cross symbols denote inhibition. Created in BioRender. Song, J. (2026), https://BioRender.com/jof4w1z, accessed on 16 May 2026.

Platelets express abundant levels of the glycoprotein CD36, which is essential for the emergence of a prothrombotic phenotype under hyperlipidaemic conditions [32] (Figure 4). When oxLDL binds to CD36, it triggers a rapid and specific signalling cascade that induces platelets to enter a “low threshold, high response” state prior to thrombosis. Animal studies reveal that CD36-deficient platelets fail to respond to oxLDL, and the incidence of thrombosis is significantly reduced in hyperlipidaemic mice [57]. oxLDL mediates platelet activation via multiple pathways through interaction with CD36. The oxLDL–CD36 complex induces the sequential phosphorylation of Src (Y416), Syk, and c-Jun N-terminal kinase (JNK), culminating in the surface presentation of P-selectin and activation of integrins, thus promoting thrombus formation [58]. CD36 also enhances NADPH oxidase-2 (NOX2) activity by phosphorylating extracellular signal-regulated kinase 5 (ERK5), generating ROS that further oxidize LDL, forming more oxLDL and establishing a positive feedback loop that promotes atherosclerosis [59]. Additionally, the CD36-Src complex rapidly induces RhoA-GTP formation, activating RhoA kinase (ROCK), inhibiting myosin light chain phosphatase (MLCP), and maintaining the high phosphorylation state of myosin light chains (MLC). Phosphorylated MLC induces platelet contraction, pseudopod extension, and the secretion of ADP, thromboxane A_2_ (TXA_2_), P-selectin, and other mediators from α-granules and dense granules, amplifying aggregation and thrombosis [60].

In summary, CD36 promotes foam cell formation in macrophages through the internalization of oxLDL and induces the secretion of a series of pro-inflammatory mediators, establishing a positive feedback loop that drives atherosclerosis. In platelets, CD36 participates in platelet stimulation and promotes thrombosis. Advances in knowledge regarding the molecular mechanisms underlying CD36’s role in atherothrombosis emphasize its potential as a candidate target for treatment and prevention.

### 3.3. CD36 and Myocardial Ischaemia–Reperfusion

Cardiac ischaemia–reperfusion injury (IRI) involves metabolic disorders and inflammatory response processes. It starts with the abrupt cessation of metabolic activities due to the blockage of coronary arteries by thrombi, which causes ischaemia [61]. During reperfusion, restoration of blood flow through thrombolysis, interventional procedures, or bypass surgery triggers specific inflammatory and oxidative stress responses, leading to aggravated microvascular damage. It eventually results in an expanded myocardial infarction area, arrhythmias, and other complications, hence impairing the recovery of cardiac function [62]. At present, clinical strategies focus more on mechanical intervention as a way of reducing injury and lack specific pharmacological interventions that can be used to treat myocardial IRI [63].

**Figure 5:**
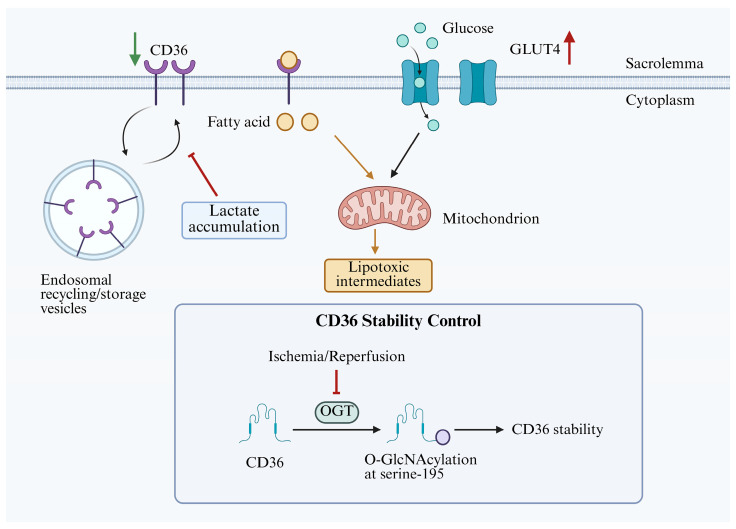
The regulation of membrane translocation and stability of CD36 during myocardial ischaemia–reperfusion. CD36 is present in at least two functionally distinct regions: the extracellular outer pool on the cell membrane and the intracellular storage/recycling pool within the cell. During ischaemia, the accumulation of lactic acid decreases intracellular pH, thereby inhibiting the membrane translocation of CD36 and upregulating GLUT4 on the sarcolemma. The O-GlcNAcylation of CD36 at the serine 195 site is catalysed by the OGT, which significantly enhances the stability of the CD36 protein. CD36—cluster of differentiation 36; GLUT4—glucose transporter type 4; OGT—O-linked β-N-acetylglucosamine transferase. The red upward arrows denote increases, the green downward arrows indicate decreases, and the cross symbols denote inhibition. Created in BioRender. Song, J. (2026) https://BioRender.com/parswgd, accessed on 14 May 2026.

In cardiomyocytes, CD36 plays a complex and multifaceted role in the process of cardiac ischaemia–reperfusion. Research indicates that lactate accumulation during ischaemia decreases intracellular pH, which inhibits the membrane translocation of CD36 [64]. Downregulation of sarcolemma CD36, accompanied by upregulation of the glucose transporter GLUT4, decreases fatty acid internalization while preventing intracellular buildup of lipotoxic intermediates and promotes glucose oxidation, hence improving energy generation and cardiomyocyte preservation (Figure 5). During reperfusion, CD36 remains at a relatively low expression level, which reduces ROS generation, attenuates oxidative stress, and facilitates functional recovery. These findings suggest that the translocation of CD36 from the cardiomyocyte sarcolemma during ischaemia–reperfusion represents an adaptive self-protective mechanism. A study published in 2025 first revealed that O-GlcNAcylation of CD36 at serine 195, catalysed by O-linked β-N-acetylglucosamine (O-GlcNAc) transferase (OGT), significantly enhances CD36 protein stability and inhibits its degradation [65]. In rats subjected to myocardial ischaemia–reperfusion and in H9C2 cells undergoing hypoxia/reoxygenation, CD36 protein levels were decreased, whereas CD36 mRNA levels remained largely unchanged. Meanwhile, OGT expression and global O-GlcNAcylation levels were also reduced. Overexpression of either OGT or CD36 promoted cardiomyocyte proliferation and inhibited apoptosis, thereby limiting the extent of myocardial infarction and enhancing cardiac performance. These apparently divergent findings may be reconciled by considering CD36 as a dynamically compartmentalized protein rather than as a single uniform pool. In cardiomyocytes, CD36 exists in at least two functionally distinct compartments: a sarcolemmal pool and intracellular storage/recycling pools, mainly associated with endosomal vesicles [66]. The sarcolemmal pool directly controls the entry of long-chain fatty acids and oxidized lipids into cardiomyocytes, whereas the intracellular pool provides a reserve for regulated trafficking and may also contribute to broader cellular homeostasis. Therefore, changes in sarcolemmal CD36 abundance and changes in total cellular CD36 protein level are not necessarily equivalent biological events. Although a transient reduction in sarcolemmal CD36 during ischaemia–reperfusion may alleviate myocardial injury by limiting fatty acid and oxLDL uptake, an excessive reduction in total cellular CD36 caused by impaired O-GlcNAc-dependent stabilization may be detrimental by depleting the intracellular recycling/reserve pool and compromising cardiomyocyte survival. Thus, selectively reducing sarcolemma CD36 while maintaining or increasing intracellular CD36 levels may represent a more effective strategy to harness its protective effects against ischaemia–reperfusion-induced myocardial injury.

In macrophages, CD36 is involved in the phagocytosis of apoptotic cells (efferocytosis), which contributes to resolving inflammation [67]. Reduced CD36 expression in macrophages negatively affects the repair phase following myocardial infarction. CD36 deficiency downregulates myeloid epithelial reproductive receptor tyrosine kinase (Mertk) and nuclear receptor subfamily 4, group A, member 1 (Nr4a1), impairing the clearance of apoptotic cells and exacerbating inflammatory responses, thereby increasing the risk of ventricular rupture. Therefore, loss of CD36 function in macrophages aggravates cardiac IRI.

Overall, CD36-mediated pathways in myocardial ischaemia–reperfusion injury are highly complex, as its functions vary across distinct cell populations and even distinct subcellular compartments within the same cell type. Consequently, a systemic enhancement or inhibition of CD36 expression is unlikely to be an effective therapeutic strategy. Instead, precise, cell-type-specific modulation of CD36 could provide a promising approach to mitigating myocardial IRI.

### 3.4. CD36 and Aortic Dissection

Aortic dissection is an extremely severe cardiovascular disease characterized by rapid progression and a generally poor prognosis. Abnormal blood flow from the true lumen toward the false lumen may result in clinical manifestations of ischaemia, as well as complications such as insufficient perfusion of vital organs, impaired aortic valve function, and pericardial tamponade. Without timely treatment, these conditions may lead to sudden aortic rupture, circulatory failure, and death in a substantial proportion of patients [68]. Despite continuous advances in surgical techniques, mortality and postoperative complication rates remain high [69].

A study on acute type A aortic dissection (ATAAD) and T cells showed that patients with aortic dissection have elevated levels of low-density lipoprotein cholesterol and palmitic acid (PA) in peripheral blood, which induce a 2- to 3-fold increase in CD36 expression on CD4^+^ T lymphocytes [70]. CD36 enhances cellular sensitivity to ferroptosis by promoting the uptake of saturated fatty acids such as PA, thereby reducing T cell number and functional impairment. This is manifested by decreased cell viability, reduced expression of activation markers, and increased programmed cell death protein 1 expression. Blocking CD36 or inhibiting ferroptosis can reverse these deleterious effects.

Currently, relatively few studies have explored the connection between CD36 and ATAAD. In the context of aortic dissection, the role of CD36 in other cell types still requires further investigation. The “CD36–fatty acid–ferroptosis–T cell functional suppression” axis also provides a novel perspective in exploring the role of CD36 in T cells. As a potential therapeutic target in ATAAD, CD36 warrants further investigation.

### 3.5. CD36 and Abdominal Aortic Aneurysm

Abdominal aortic aneurysm (AAA) is a severe vascular condition marked by persistent inflammation of the vessel wall and extracellular matrix remodelling, which together drive the progressive dilation of the abdominal aorta [71]. With population ageing, the incidence of AAA shows a growing trend and constitutes a major contributor to death in older individuals [72]. AAA rupture is a catastrophic event associated with an extremely high mortality rate of up to 80% [73]. At present, no effective pharmacological therapy is available to prevent aneurysm expansion, and surgical procedures are still the only effective treatment.

AAA may develop because of atherosclerosis, in which CD36 serves as a key mediator of foam cell formation and inflammatory responses during atherosclerotic plaque development. A study of Salmonella-induced infectious aortic aneurysms found that endothelial injury, localized inflammatory infiltration, and focal thinning of the vascular wall in the setting of atherosclerosis create favourable conditions for Salmonella infection [74]. Moreover, Salmonella infection differentially regulates CD36 expression in a plasmid-dependent manner: strains carrying plasmids downregulate CD36 expression, whereas those lacking plasmids upregulate it, thereby influencing the progression of atherosclerosis and the formation of aortic aneurysms.

Beyond its role in atherosclerosis, CD36 is involved in additional pathogenic pathways that contribute to aortic wall injury and promote the formation and evolution of AAA. Investigations reveal that haematopoietic and vascular cells in patients with AAA respond to oxidized lipids through CD36, which downregulates key antioxidant factors and leads to ROS accumulation [72]. Compared with healthy abdominal aortas, CD36 mRNA levels are elevated in aneurysmal vascular walls and are mainly localized to the vascular media. However, no direct correlation has been identified between CD36 expression and aneurysm diameter. In addition, CD36 is localized to the plasma membrane of endothelial cells across the aortic wall, where it transports fatty acids [73]. In the aneurysm-associated aberrant endothelial cell subpopulation, CD36, as one of the most highly expressed genes, may contribute to lipid metabolic reprogramming in endothelial cells.

Recent studies have shown that the interaction between CD36 on red blood cells and thrombospondin-1 on platelet surfaces induces phosphatidylserine exposure on red blood cells, thereby enhancing the procoagulant activity of both cell types [71]. Patients with AAA exhibit increased CD36 exposure on the surface of red blood cells. Biomechanical stress within the aneurysmal segment enhances CD36 exocytosis on red blood cells and platelets, as well as platelet–red blood cell aggregation. Deletion of CD36 in red blood cells confers resistance to experimentally induced AAA in mice.

CD36 expression is regulated by additional proteins, such as the proprotein convertase subtilisin/kexin type 9 (PCSK9) protein. Research shows that the number of hyperplastic adipocytes is markedly increased in the adventitia of ruptured AAA, accompanied by decreased PCSK9 and increased CD36 expression, with a negative correlation between the two. Overexpression of CD36 may excessively enhance fatty acid transport into adipocytes, thereby promoting hypertrophic adipocyte accumulation and leading to degeneration of the aortic adventitia, ultimately triggering AAA rupture. Although PCSK9 inhibitors have emerged as a novel therapeutic strategy for CVD, they may not be suitable for patients at risk of AAA rupture [75].

Collectively, CD36 participates in multiple metabolic, inflammatory, and thrombotic pathways across macrophages, haematopoietic cells, endothelial cells, erythrocytes, and adipocytes, all of which are implicated in AAA development and rupture. Therefore, CD36 may represent a therapeutic target for limiting the progression and rupture of AAA.

### 3.6. CD36 and Calcific Aortic Valve Disease

Calcific aortic valve disease (CAVD) is a common degenerative disorder of the aortic valve, characterized primarily by progressive calcification of the valve leaflets. As the population ages, the global prevalence of CAVD is increasing rapidly [76]. Calcific lesions can restrict valvular motion and obstruct the left ventricular ejection pathway, thereby leading to aortic stenosis [77]. Once symptoms of aortic stenosis develop, the prognosis becomes poor. In patients aged approximately 80 years, the mortality rate is about 50% within 2 years and approximately 80% within 5 years in the absence of surgical intervention or transcatheter valve replacement [78]. Currently, no drug has been confirmed to effectively attenuate the advancement of CAVD. Moreover, interventions targeting traditional risk factors for CAVD, such as lipid-lowering therapy, have also failed to halt disease progression [79].

**Figure 6:**
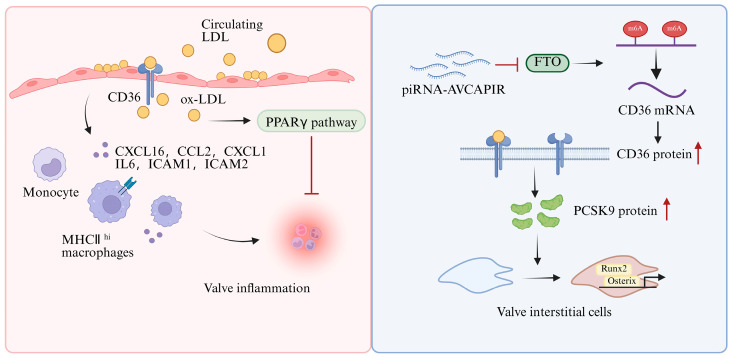
Regulatory mechanisms of CD36 in calcific aortic valve disease. In VECs, CD36 regulates inflammation and lipid deposition by activating the PPARγ pathway through LDL uptake. In VICs, piRNA-AVCAPIRs inhibit the demethylase activity of FTO, thereby enhancing CD36 stability. Upregulation of CD36 increases PCSK9 protein levels, accelerating the osteogenic transition of VICs and promoting aortic valve calcification. CD36—cluster of differentiation 36; oxLDL—oxidized low-density lipoprotein; PPARγ—peroxisome proliferator-activated receptor gamma; CXCL1—C-X-C motif chemokine ligand 1; CCL2—C-C motif chemokine ligand 2; CXCL16—C-X-C motif chemokine ligand 16; IL6—interleukin 6; ICAM1—intercellular adhesion molecule 1; ICAM2—intercellular adhesion molecule 2; piRNA-AVCAPIRs—aortic valve calcification-associated PIWI-interacting RNA; FTO—fat mass and obesity-associated protein; PCSK9—proprotein convertase subtilisin/kexin type 9; Runx2—Runt-related transcription factor 2; Osterix—Sp7 transcription factor; VECs—valvular endothelial cells; VICs—valvular interstitial cells. Cross symbols denote inhibition. Red upward arrows denote increases. Created in BioRender. Song, J. (2026), https://BioRender.com/4iefo48, accessed on 16 May 2026.

In valvular endothelial cells (VECs), CD36 primarily contributes to early lipid deposition and inflammatory responses during CAVD (Figure 6, left). In hyperlipidaemic mice, LDL is the predominant lipoprotein that accumulates in the aortic valve, and the number of CD36^+^ endothelial cells is increased, thereby facilitating LDL uptake into the cytoplasm [80]. Within the local valvular microenvironment, LDL is further oxidized to oxLDL, which activates PPARγ and subsequently restrains inflammatory progression. By contrast, inhibition of PPARγ dramatically elevates the level of inflammatory cytokines and cell adhesion molecules, including C-X-C motif chemokine ligand (CXCL) 1, C-C motif chemokine ligand 2 (CCL2), CXCL16, IL6, intercellular adhesion molecule (ICAM) 1, and ICAM2. This promotes the recruitment of monocyte-derived MHC-II^hi^ macrophages with pro-inflammatory properties. An imbalance between these anti-inflammatory and pro-inflammatory responses may further amplify valvular inflammation.

In valvular interstitial cells (VICs), increased CD36 expression promotes the progression of valvular calcification (Figure 6, right). During aortic valve calcification, aortic valve calcification-associated PIWI-interacting RNA (piRNA-AVCAPIR) is markedly upregulated and directly interacts with fat mass and obesity-associated protein (FTO), thereby inhibiting its N6-methyladenosine demethylase activity [81]. As a result, AVCAPIR prevents FTO-mediated demethylation of CD36 mRNA and enhances its stability. The increased AVCAPIR-dependent CD36 protein further upregulated PCSK9 protein, thereby accelerating the osteogenic transformation of aortic VICs and promoting aortic valve calcification [81,82]. Correspondingly, the abundance of osteogenic differentiation markers, including Runt-related transcription factor 2 (Runx2) and Sp7 transcription factor (Osterix), is significantly reduced in the CD36/ApoE DKO aortic valves, accompanied by attenuation of calcified nodule formation, calcium deposition, and ROS generation.

Collectively, CD36 is a critical mediator in aortic valve calcification, contributing not only to early lipid deposition and inflammatory initiation but also to the osteogenic differentiation of VICs during disease progression. Therefore, CD36 may represent a promising therapeutic target for the prevention and treatment of aortic valve calcification. Nevertheless, the mechanisms by which CD36 participates in valvular calcification remain to be further elucidated, and its potential role in other cardiovascular valvular calcification warrants further investigation.

### 3.7. CD36 and Heart Failure

HF is a clinical syndrome caused by structural and/or functional abnormalities of the heart and is accompanied by profound metabolic remodelling. This includes reduced fatty acid oxidative capacity and a mismatch between lipid uptake and utilization, which, in turn, leads to toxic lipid accumulation, mitochondrial dysfunction, fibrosis, and impaired contractility [83,84]. HF represents a major global health burden; although its incidence has stabilized in some developed countries, the overall prevalence of HF continues to rise owing to population ageing and improved survival of affected patients [85]. Regarding treatment, in HF with reduced ejection fraction (HFrEF), combined neurohormonal blockade and sodium–glucose cotransporter 2 (SGLT2) inhibitor therapy has substantially improved survival and reduced hospitalization [86]. By contrast, therapeutic benefits in HF with preserved ejection fraction (HFpEF) remain relatively limited and are mainly derived from SGLT2 inhibitors, diuretic therapy, and the management of comorbidities [87]. Device therapy, left ventricular assist devices (LVADs), and heart transplantation provide important treatment options for selected patients; however, their implementation is still restricted by factors such as treatment tolerance and cost [88,89]. As a major facilitator of LCFA uptake in cardiomyocytes, CD36 is located at the crossroads of substrate supply and energy production [90,91]. Therefore, elucidating how CD36 contributes to the development and progression of HF may provide new mechanistic insights and help identify potential therapeutic targets. In this section, we mainly summarize current findings regarding the role of CD36 in HF associated with dilated cardiomyopathy (DCM) and hypertrophic cardiomyopathy (HCM).

Regarding DCM, a nonsense variant in CD36 (*rs3211938*) has been associated with an increased risk of DCM, with homozygous carriers exhibiting approximately a threefold higher likelihood of developing the disease [24]. Notably, even in the absence of overt cardiomyopathy, these individuals show an approximately 8% reduction in left ventricular ejection fraction. Mechanistically, loss of CD36 function in human induced pluripotent stem cell-derived cardiomyocytes reduces fatty acid uptake, disrupts cardiac metabolism, and impairs contractility, thereby supporting a causal link between defective CD36-mediated lipid handling, impaired myocardial energetics, and systolic dysfunction. A 2025 study further demonstrated that CD36 protein abundance was decreased in myocardial tissue from patients with DCM, in the hearts of transverse aortic constriction (TAC) mice, and in hypertrophic cardiomyocytes, accompanied by attenuation of insulin receptor/Akt signalling [92]. In the same study, cardiomyocyte-specific overexpression of CD36 improved left ventricular systolic function and attenuated hypertrophy and fibrosis in pressure-overloaded hearts. Although these experimental systems do not encompass all aetiologies of DCM, they support the concept that reduced CD36 expression in the failing myocardium exacerbates metabolic inflexibility and accelerates the progression to HF when energy supply becomes limiting.

Studies on HCM have shown that cardiomyocyte-specific deletion of CD36 accelerates the transition from compensated hypertrophy to systolic HF [93]. Similarly, global CD36 deficiency aggravates TAC-induced cardiac dysfunction, mortality, hypertrophy, and interstitial fibrosis, and it is accompanied by depletion of tricarboxylic acid cycle intermediates and high-energy phosphates despite increased glucose utilization [94]. Notably, supplementation with medium-chain fatty acids partially rescues cardiac dysfunction, indicating that the adverse phenotype is primarily driven by insufficient energy production secondary to impaired long-chain fatty acid uptake. However, CD36 appears to exert a biphasic effect in hypertrophic and failing hearts. Under combined metabolic and mechanical stress, such as a Western diet with pressure overload, CD36 deficiency protects against diet-associated exacerbation of cardiac remodelling and dysfunction [95]. More recent evidence suggests that in contrast to complete knockout, partial cardiac knockdown of CD36 reduces toxic lipid accumulation, oxidative stress, hypertrophy, and fibrosis while preserving ATP production by increasing glucose flux in the tricarboxylic acid cycle [96]. Taken together, these findings indicate that insufficient CD36 may starve the pressure-overloaded heart, whereas excessive or mislocalized CD36 in a lipid-rich environment may promote lipotoxic remodelling.

Overall, current evidence indicates that CD36 is not a simple “on/off” therapeutic target in HF. In DCM, insufficient CD36 function may impair myocardial energetics and promote systolic dysfunction, whereas in hypertrophic remodelling, its effects appear to depend on the balance between energy demand and lipid excess. From a therapeutic perspective, restoring an appropriate level and subcellular distribution of CD36, rather than uniformly inhibiting or enhancing its activity, may represent a more rational strategy to prevent progression from metabolic remodelling to overt HF.

### 3.8. Cardiovascular Complications of Diabetes

Diabetes is a chronic metabolic disorder arising from the combined effects of hereditary and external factors [97]. Persistent hyperglycaemia causes constant damage to blood vessels and nerves throughout the body, and it is a significant risk factor for disability and death in people with diabetes. The modern state of diabetes management remains concerning. The percentage of patients with diabetes receiving treatment in China who have achieved adequate glycaemic control is merely 42.0% [98]. Cardiovascular complications of diabetes include CAD, stroke, peripheral artery disease, and diabetic cardiomyopathy [99]. These complications share common pathogenic mechanisms, including hyperglycaemia-induced oxidative stress, chronic inflammatory responses, and endothelial dysfunction.

CD36 is a key molecule in type 2 diabetes and its cardiovascular complications, but its role in type 1 diabetes remains incompletely understood. In patients with prediabetes, the expression of CD36 increases, which may disrupt the balance between Th17 cells and Treg cells and thereby promote inflammatory responses [100,101]. CD36 has been implicated in multiple aspects of diabetes progression, including insulin resistance [102], pancreatic β-cell dysfunction, and the loss of β-cell mass, all of which eventually disrupt insulin secretion [103,104]. Reduced glucose metabolism secondary to insulin resistance promotes a metabolic switch toward fatty acid β-oxidation. This process is accompanied by the translocation of CD36 to the myocyte membrane, resulting in enhanced uptake of LCFAs in cardiomyocytes and contributing to lipotoxic cardiomyopathy in patients with diabetes [105]. In the initial stage of diabetes, elevated insulin levels promote CD36 mRNA expression by activating the transcription factor forkhead box protein O1 (FOXO1) and promote CD36 translocation to the sarcolemma via the phosphatidylinositol 3-kinase-Akt (PI3K-Akt) signalling pathway [106]. In the later stages of diabetes, elevated blood glucose and triglycerides further increase CD36 expression and its localization on the sarcolemma. Additionally, diabetes-induced alterations in microRNAs, including upregulation of miR-320 and downregulation of miR-200b-3p, promote CD36 transcription and translation, further facilitating its sarcolemma distribution [107]. Moreover, the accumulation of LCFAs induced by CD36 activation activates PPARs, which further upregulate CD36’s transcription and localization on the sarcolemma. This establishes a positive feedback loop that exacerbates fatty acid uptake and lipotoxicity in the diabetic myocardium [108].

Collectively, beyond its metabolic functions in glucose and lipid utilization, CD36 also contributes to immune regulation, making it not only a key mediator in the pathogenesis of diabetes and its associated complications but also a potentially valuable therapeutic target for clinical intervention.

Based on the above discussions, Table 1 summarizes the roles of CD36-mediated inflammatory pathways and lipid metabolism in the development and progression of CVDs and cardiovascular complications associated with inflammatory disorders.

## 4. Targeting CD36 in the Treatment of Cardiovascular Diseases

Currently, various therapeutic strategies targeting CD36 have been explored for the treatment of CVD. These approaches include small-molecule inhibitors, natural products or herbal components, monoclonal antibodies, and gene therapy. Collectively, these strategies improve vascular function primarily by directly or indirectly inhibiting CD36 expression and activity, thereby modulating lipid uptake, suppressing inflammatory responses, and blocking related signalling pathways.

Small-molecule inhibitors mainly exert their effects by interfering with the modification or function of CD36. 2-Bromopalmitic acid (2-bp) is a palmitoylation inhibitor that suppresses the palmitoylation of CD36 [109]. Palmitoylation is crucial for the membrane localization and functional activity of CD36 [35]. Studies have shown that in an ischemic cardiomyocyte model, treatment with 2-bp (25 μM; 24 h) inhibits CD36 palmitoylation, thereby reducing its membrane localization, decreasing fatty acid uptake, and alleviating the accumulation of lipid toxicity metabolites, such as ceramides and diacylglycerol, ultimately improving mitochondrial function. Common lipid-lowering drugs, such as simvastatin, are small-molecule statins. A study on ApoE^−/−^ mice showed that simvastatin attenuated atherosclerotic lesions, reduced serum IL-6 and TNF-α levels, and downregulated aortic CD36 expression, suggesting that the anti-atherosclerotic and anti-inflammatory effects of statins may be mediated, at least in part, through modulation of the CD36 pathway [110].

GLP-1 receptor agonists (GLP-1RAs), such as liraglutide and semaglutide, exert beneficial effects on glycaemic control and cardiovascular risk reduction. Accumulating evidence suggests that GLP-1RAs may improve cardiometabolic homeostasis through multiple signalling pathways. Notably, liraglutide has been reported to activate the CD36 downstream JNK-AP-1 pathway in high-glucose-stimulated cardiac fibroblasts, thereby upregulating CD36 expression and mediating associated anti-fibrotic effects [111]. These findings indicate that GLP-1RAs may indirectly influence myocardial fibrosis and inflammatory processes by modulating CD36-related signalling transduction.

SGLT2 inhibitors, including empagliflozin and dapagliflozin, have well-established cardiovascular benefits. Animal studies have shown that empagliflozin can activate AMPK signalling; reduce the expression of lipotoxicity-associated proteins, including CD36; and decrease fatty acid accumulation in the myocardium, thereby improving cardiac metabolism [112]. Specifically, empagliflozin treatment has been reported to significantly downregulate CD36 levels in cardiac tissues, accompanied by reduced lipotoxic intermediates and enhanced autophagy. Therefore, beyond their glucose-lowering effects, SGLT2 inhibitors may confer additional cardioprotective effects by suppressing CD36-mediated fatty acid uptake pathways.

Members of the PPAR family regulate the expression of genes involved in lipid metabolism, and PPARγ and PPARα have been shown to transcriptionally upregulate CD36. PPARγ agonists, including thiazolidinediones such as rosiglitazone and pioglitazone, markedly enhance CD36 expression in adipocytes and macrophages, thereby promoting fatty acid uptake. In obesity and diabetes models, previous studies have reported that PPARγ activation is co-expressed with CD36, forming a positive regulatory loop that facilitates lipid uptake and storage [113]. Similarly, PPARα agonists, such as fibrates (including fenofibrate), can also increase CD36 expression. Experimental studies have shown that fenofibrate treatment significantly increases CD36 mRNA expression in the adipose tissue of hypercholesterolaemic rabbits and markedly enhances the uptake and degradation of oxLDL [114]. Clinically, PPAR agonists are widely used to treat dyslipidaemia and metabolic syndrome because of their beneficial effects on lipid metabolism and insulin sensitivity. However, under metabolically stressed conditions such as obesity or diabetes, the CD36-upregulating effect of these agents may aggravate lipid overload in cardiomyocytes and macrophages [32]. Therefore, the therapeutic benefits of PPAR agonists should be carefully balanced against their potential cardiovascular risks.

With the continuing advancement of research on traditional Chinese medicine components and their mechanisms of action, an increasing number of natural substances and herbal components have been found to improve lipid metabolism and attenuate inflammatory responses by regulating CD36 and its related signalling pathways. For example, protocatechuic acid, the main metabolite of anthocyanins in vivo, can enhance the expression and activity of antioxidant enzymes and promote mitochondrial biogenesis by regulating the CD36/AMPK signalling pathway, thereby ameliorating high-fat-induced oxidative damage in vascular endothelial cells [115]. In addition, Baoyuan Decoction, a traditional Chinese medicine formula with complex ingredients, exerts its effects through multiple targets and pathways. Recent studies have confirmed that it can inhibit lipid peroxidation-mediated CD36 expression and the Src/MKK4/JNK pathway, thereby reducing macrophage foam cell formation and exerting anti-atherosclerotic effects [116]. Danshensu (DSS)—one of the major water-soluble bioactive constituents of Salvia miltiorrhiza Bunge—has shown considerable cardioprotective potential. Previous studies have demonstrated that in a RAW264.7 macrophage-derived foam cell model, DSS significantly downregulated the expression of CD36 and its homologous receptor scavenger receptor BI (SR-BI), thereby reducing oxLDL uptake by macrophages [117]. Meanwhile, DSS upregulated ATP-binding cassette transporter A1 (ABCA1) and ATP-binding cassette transporter G1 (ABCG1) expression, promoted cholesterol efflux, and consequently, inhibited foam cell formation and intracellular lipid accumulation. Recent evidence indicates that sodium danshensu (SDSS) can stabilize vulnerable atherosclerotic plaques by suppressing macrophage-mediated inflammation and targeting the IKKβ/NF-κB signalling pathway [118]. Whether CD36 is involved in this inflammation-related pathway remains to be further investigated.

Monoclonal antibodies are widely used in the medical field due to their high specificity, efficiency, safety, and strong design flexibility. At present, monoclonal antibodies targeting CD36 are mainly used for CD36 detection and cell sorting [119]. Nanotechnology offers precise drug delivery and the advantage of reducing systemic side effects. In recent years, the integration of CD36 monoclonal antibodies with nanotechnology-based targeted delivery systems has provided a new direction for the treatment of CD36-related diseases. A team led by Professor Shuai Xintao from Sun Yat-sen University designed a cationic polymer carrier composed of polyethylene glycol–polyaspartic acid-grafted polyethyleneimine (mPEG-PAsp-(g-PEI)) and modified its surface with the single-chain antibody of CD36 [120]. This carrier can encapsulate siRNA targeting the p21-activated kinase 1 gene (siPAK1) and specifically recognize and bind to macrophages with high CD36 expression within plaques, thereby enabling targeted delivery of siRNA. After entering the cells, siPAK1 effectively silences PAK1 expression, thereby downregulating its downstream inflammatory mediator monocyte chemoattractant protein-1 (MCP-1) and IL-6, reducing foam cell formation and ultimately alleviating the pathological process of atherosclerosis. This research provides an efficient and targeted gene therapy strategy for the treatment of atherosclerosis. However, this technology is still in the animal experimentation stage, and its application in clinical trials will require multiple rounds of rigorous testing to ensure safety.

In addition to conventional monoclonal antibodies, anti-CD36 single-chain variable fragments (scFvs) have also demonstrated therapeutic potential. A recent study identified a fully human scFv, D11, which effectively blocked CD36 in macrophage-like THP-1 cells, thereby reducing oxLDL uptake, suppressing the formation of the foam cell phenotype, and decreasing lipid droplet accumulation, as well as the expression of lipid metabolism-related genes [121]. Although the current evidence remains limited to in vitro studies, and substantial time and further efforts will be required to advance in vivo investigations and clinical trials, this finding provides a promising direction for the development of therapeutic agents targeting CD36 based on scFv D11.

Circulating levels of cell-free soluble CD36 are significantly elevated in patients with atherosclerosis and concomitant metabolic disorders. A multicentre cohort study confirmed that the serum soluble CD36 (sCD36) levels are independently positively associated with carotid artery ultrasound intima–media thickness, PET-CT inflammatory signals, and the incidence of major adverse cardiovascular events [42]. In the future, sCD36 may be incorporated into cardiovascular disease risk stratification models or used as a novel pharmacodynamic indicator for targeted CD36 therapy.

Given the dual and complex physiological functions of CD36, drug development in this field faces substantial challenges. Complete inhibition of CD36 is not a feasible therapeutic strategy. Therefore, current research is still focused on precisely regulating CD36 functions. Future studies should expand the application of nanotechnology combined with monoclonal antibodies for targeted delivery and develop more specific targeting strategies for antibody-conjugated drugs. In addition, combination therapy may represent a promising direction where CD36 inhibitors are used with hypolipidemic agents, anti-inflammatory drugs, or antiplatelet drugs. Such strategies may produce synergistic effects, reduce the required dose of each agent, and thereby minimize side effects.

## 5. Conclusions and Perspective

Current evidence indicates that CD36 is not merely a fatty acid transporter but also a key factor linking lipid metabolism, inflammatory responses, and related biological processes. CD36 is implicated in multiple types of CVDs, but the strength of current evidence varies markedly among different cardiovascular subtypes. The mechanisms by which CD36-mediated inflammatory responses and lipid metabolic dysregulation contribute to atherosclerosis have been relatively well characterized. In thrombosis, CAVD, and AAA, several pathways associated with disease progression have been identified, although detailed mechanistic networks remain incompletely established. In hypertension, the mechanistic link by which CD36 mediates non-alcoholic steatohepatitis-induced hypertension is relatively clear, whereas how CD36 interacts with Ang II to influence blood pressure requires further investigation. In myocardial IRI, aortic dissection, HF, and other cardiovascular conditions, current studies have mainly demonstrated associations with CD36 and identified several CD36-interacting factors, while the complete mechanistic cascades remain to be clarified. For example, in myocardial IRI, defining how intracellular storage/recycling compartments of CD36 coordinate with CD36 translocation to the plasma membrane and thereby influence cellular homeostasis is important for understanding how CD36 may be targeted to modulate myocardial ischaemia–reperfusion injury.

In addition, patients with systemic inflammatory diseases may have an increased incidence of CVDs, and in many cases, these cardiovascular complications represent a major cause of mortality [122,123,124,125,126,127]. Therefore, the role of CD36 in inflammatory diseases still has considerable research potential. Among systemic inflammatory diseases, cardiovascular complications of diabetes have been extensively studied, and multiple mechanistic pathways have been identified in cardiomyocytes. In this context, how CD36 affects the imbalance of Th17/Treg cells and thereby promotes inflammatory progression is also worthy of further investigation. Moreover, systemic lupus erythematosus (SLE) and inflammatory bowel disease (IBD) are associated with an increased risk of CVDs, raising the question of whether CD36 may serve as a key mediator linking SLE or IBD to cardiovascular pathology. Previous studies have shown that, in some cases of SLE, impaired CD36-mediated monocyte efferocytosis delays the clearance of apoptotic cells and eventually leads to secondary necrosis, which is a prerequisite for autoantigen exposure and immune complex formation in SLE [128]. In IBD, palmitic acid enters cells via CD36 and undergoes palmitoylation-mediated modification, thereby participating in and enhancing the activation of the signal transducer and activator of transcription 3 (STAT3) signalling pathway [129]. Sustained activation of this pathway may aggravate intestinal and systemic inflammation and promote IBD progression. Mechanistically, it can be inferred that, under the inflammatory environment of SLE and IBD, LDL may be more prone to oxidation into oxLDL [130], which is then taken up by macrophages via CD36 and promotes foam cell formation; however, this process has not yet been directly verified. In addition, increased CD36 expression on platelets from patients with IBD is associated with an elevated risk of thromboembolism [131,132]. This may be related to CD36-mediated platelet activation, inflammatory responses, and insulin resistance. The specific inflammatory pathways involved, as well as the relationship between insulin resistance and thromboembolism, remain interesting directions for future research.

Overall, the role of CD36 in chronic inflammation is highly complex. Current studies mainly focus on its pro-inflammatory effects, and relatively few have investigated its anti-inflammatory actions mediated by the uptake of other ligands. Existing evidence suggests that, in the later stages of stroke, CD36-dependent pathways may limit or resolve inflammation [133], although the underlying mechanisms remain unclear. Future studies should systematically investigate and clarify the binding sites, signalling duration, and competitive interactions between pro-inflammatory and inflammation-resolving ligands of CD36. Such efforts will further enrich our understanding of the mechanisms by which CD36 participates in CVDs and may facilitate more precise modulation of CD36.

Current therapeutic strategies targeting CD36 are mechanistically diverse, including GLP-1RAs and SGLT2 inhibitors; PPAR agonists; small-molecule inhibitors; natural compounds, such as active ingredients derived from traditional Chinese medicine; biologics; and specific CD36 antagonists. However, most candidate agents remain at the preclinical stage. Before clinical translation, CD36-targeted candidate therapies should meet several requirements. First, they should modulate CD36 in a cell-type- or pathway-selective manner and exhibit a favourable safety profile, rather than causing broad and systemic CD36 blockade. Second, their efficacy should be demonstrated in available human cell models, organ cultures, or tissue models, as well as in multiple animal models that reproduce the metabolic and inflammatory microenvironment of the target disease. For CD36-targeted therapies, pharmacodynamic biomarkers should be clearly defined—such as membrane CD36 abundance in target cells, oxLDL uptake, inflammatory signalling markers, lipid accumulation, platelet activation indicators, and sCD36—to facilitate patient selection and dose optimization. Most importantly, their benefits should be evaluated against current standard therapies and preferably assessed in combination with these standard treatments to demonstrate their added value and translational potential. Finally, for antibodies, nanoparticles, and gene therapies, properties such as targeted delivery, reversibility, immunogenicity, long-term toxicity, and manufacturability must be rigorously evaluated.

## Figures and Tables

**Table 1:** Role of CD36 in cardiovascular diseases and cardiovascular complications associated with inflammatory disorders.

Disease	Major Cell Types/Tissues Involved	Signalling Pathway	Net Effect	Pathophysiological Consequence
Hypertension	Liver tissues	PDE4-CD36-TGF-β1	Vascular inflammation and fibrosis	Elevated blood pressure
	Endothelial cells	Ang II-CD36-ferroptosis	Disrupted integrity of endothelial cells and decreased NO	
Atherosclerosis	Macrophages	oxLDL uptake-TLR4/6-NF-κB/MAPK/NLRP3/ PPARγ/FAO/AMPK	Formation of foam cells and inflammatory environment	Formation and development of atherosclerotic plaques
Thrombosis	Platelets	CD36-Src-Syk-JNK	Presentation of P-selectin and activation of integrins	Thrombosis
		CD36-ERK5-NOX2	More oxLDL	
		CD36-Src-RhoA-GTP-ROCK-MLCP-MLC	Platelets contract, pseudopodia extend, α granules and dense granules secrete substances	
Myocardial IRI	Cardiomyocytes	CD36 O-GlcNAcylation	CD36 membrane translocation	The function is cell-specific
	Macrophages	Mertk/Nr4a1	Apoptotic cell clearance and inflammation resolution	
Aortic dissection	CD4+ T cells	CD36–fatty acid– ferroptosis–T cell	Decrease in T cell quantity and dysfunction	T cell dysfunction with poor prognosis
AAA	Haematopoietic cells and vascular cells	CD36-oxidized lipids	ROS accumulation	Formation and rupture of AAA
	Erythrocyte and platelets	CD36–thrombospondin-1	Procoagulant	
	Adipocytes	PCSK9-CD36	Hypertrophic adipocyte accumulation	
CAVD	VECs	CD36-oxLDL-PPARγ	Lipid deposition/antiinflammation	Anti-inflammatory/pro-inflammatory responses
	VICs	piRNA-AVCAPIRs-FTO-CD36-PCSK9-osteogenic differentiation	Aortic valve calcification	Formation of calcified nodules
HF	Cardiomyocytes	DCM: rs3211938/insulin receptor-Akt signalling	Myocardial energy metabolism and contraction function	In DCM and HCM, complete knockout of CD36 accelerates HF
		HCM: Unknown		
Diabetes	Th17/Treg cells	Unknown	Pro-inflammation	Promotes diabetes and cardiovascular complications
	Cardiomyocytes	FOXO1-CD36-PI3K-Akt miR-320/miR-200b-3p-CD36 CD36-LCFAs-PPARs-CD36	Lipid intake and lipid toxicity	

## Data Availability

No new data were created or analyzed in this study. Data sharing is not applicable to this article.

## Abbreviations

2-bp	2-Bromopalmitic acid
AAA	Abdominal aortic aneurysm
ABCA1	ATP-binding cassette transporter A1
ABCG1	ATP-binding cassette transporter G1
ACEIs	Angiotensin-converting enzyme inhibitors
AMPK	AMP-activated protein kinase
Ang II	Angiotensin II
AP-1	Activator protein 1
ApoE^−/−^	Apolipoprotein E gene-deficient
ARBs	Angiotensin II receptor blockers
ATAAD	Acute type A aortic dissection
CAD	Coronary artery disease
CAVD	Calcific aortic valve disease
CCBs	Calcium channel blockers
CCL2	C-C motif chemokine ligand 2
CD36	Cluster of differentiation 36
CHD	Coronary heart disease
CVDs	Cardiovascular diseases
CXCL	C-X-C motif chemokine ligand
DAMPs	Damage-associated molecular patterns
DCM	Dilated cardiomyopathy
DSS	Danshensu
EOCAD	Early-onset coronary artery disease
ERK5	Extracellular signal-regulated kinase 5
FAO	Fatty acid oxidation
FOXO1	Forkhead box protein O1
FTO	Fat mass and obesity-associated protein
HCM	Hypertrophic cardiomyopathy
HF	Heart failure
HFpEF	Heart failure with preserved ejection fraction
HFrEF	Heart failure with reduced ejection fraction
IBD	Inflammatory bowel disease
ICAM	Intercellular adhesion molecule
IL	Interleukin
IRFs	Interferon regulatory factors
IRI	Ischaemia–reperfusion injury
JNK	c-Jun N-terminal kinase
LCFAs	Long-chain fatty acids
LVADs	Left ventricular assist devices
MAPK	Mitogen-activated protein kinase
MCP-1	Monocyte chemoattractant protein-1
Mertk	Myeloid epithelial reproductive receptor tyrosine kinase
MLCP	Myosin light chain phosphatase
mPEG-PAsp-(g-PEI)	Polyethylene glycol–polyaspartic acid grafted polyethyleneimine
MRAs	Mineralocorticoid receptor antagonists
MyD88	Myeloid differentiation primary response protein 88
NAFLD	Non-alcoholic fatty liver disease
NF-κB	Nuclear factor κB
NLRP3	NOD-like receptor pyrin domain-containing 3
NO	Nitric oxide
NOX2	NADPH oxidase-2
Nr4a1	Nuclear receptor subfamily 4, group A, member 1
O-GlcNAc	O-linked β-N-acetylglucosamine
OGT	O-linked β-N-acetylglucosamine transferase
Osterix	Sp7 transcription factor
oxLDL	Oxidized low-density lipoprotein
PA	Palmitic acid
PAMPs	Pathogen-associated molecular patterns
PCSK9	Proprotein convertase subtilisin/kexin type 9
PDE4	Phosphodiesterase 4
PI3K-Akt	Phosphatidylinositol 3-kinase-Akt
piRNA-AVCAPIR	Aortic valve calcification-associated PIWI-interacting RNA
PPAR	Peroxisome proliferator-activated receptor
ROCK	RhoA kinase
ROS	Reactive oxygen species
Runx2	Runt-related transcription factor 2
sCD36	Soluble CD36
scFvs	Single-chain variable fragments
SDSS	Sodium danshensu
SGLT2	Sodium–glucose cotransporter 2
siPAK1	siRNA targeting the p21-activated kinase 1 gene
SLE	Systemic lupus erythematosus
SR-BI	Scavenger receptor BI
STAT3	Signal transducer and activator of transcription 3
T2DM	Type 2 diabetes
TAC	Transverse aortic constriction
TGF-β1	Transforming growth factor-β1
TLR	Toll-like receptor
TNF-α	Tumour necrosis factor-α
TRIF	TIR domain-containing adaptor inducing interferon-β
TXA_2_	Thromboxane A_2_
VECs	Valvular endothelial cells
VICs	Valvular interstitial cells
WHO	World Health Organization
